# Left ventricular aneurysm following patch dehiscence after surgical repair of post-myocardial infarction ventricular septal rupture: a rare case report

**DOI:** 10.1093/ehjcr/ytaf453

**Published:** 2025-09-16

**Authors:** Nabil Laktib, Selma Saidi, Ilyasse Asfalou, Aatif Benyass, Zouhair Lakhal

**Affiliations:** Cath-Lab of the Cardiology Center, Mohammed V Military Teaching Hospital, Mohammed V University, Hay Riad, Rabat 10100, Morocco; Intensive Care Unit of the Cardiology Center, Mohammed V Military Teaching Hospital, Mohammed V University, Hay Riad, Rabat 10100, Morocco; Non-Invasive Cardiac Exploration Department, Cardiology Center, Mohammed V Military Teaching Hospital, Mohammed V University, Hay Riad, Rabat 10100, Morocco; Cardiology Center, Mohammed V Military Teaching Hospital, Mohammed V University, Hay Riad, Rabat 10100, Morocco; Cath-Lab of the Cardiology Center, Mohammed V Military Teaching Hospital, Mohammed V University, Hay Riad, Rabat 10100, Morocco

## Case description

A 63-year-old former smoker male without comorbidities was admitted 5 years ago in cardiogenic shock, 13 days after an unrecognized and untreated inferior myocardial infarction presenting as epigastralgia. Echocardiography showed basal and median inferior wall hypokinesia and a 15 mm basal ventricular septal rupture. Coronarography revealed distal left circumflex artery occlusion. He underwent infarct exclusion patch plasty through right atriotomy. Revascularization was not performed due to non-viable myocardium. At 2 weeks discharge, follow-up echocardiography showed an 8 mm left-to-right shunt and a 40 × 30 mm inferoseptal and inferior aneurysm, with a slightly impaired ejection fraction. Redo-surgery was considered but refused. The patient remained asymptomatic under surveillance.

**Figure 1:**
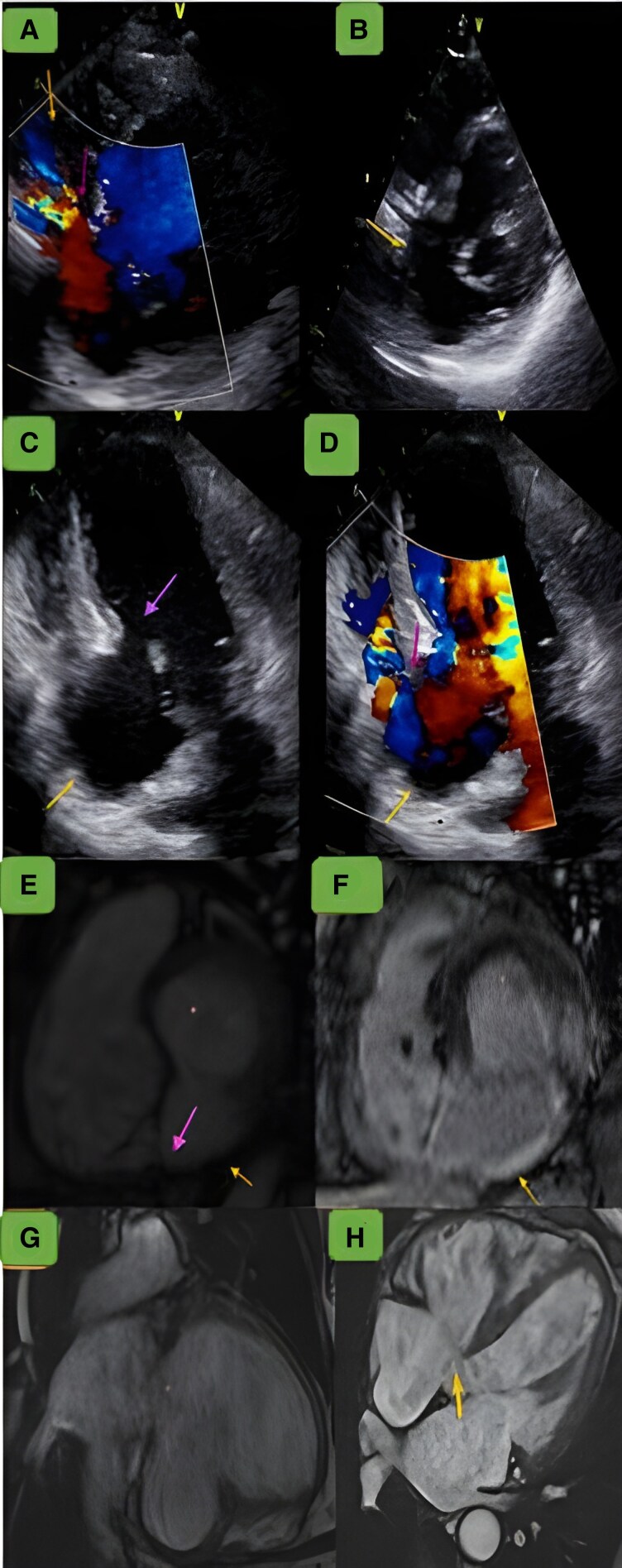
(*A*) The parasternal short-axis view with colour Doppler imaging demonstrates the aneurysm (upper arrow) in the basal inferior and inferoseptal walls, appearing as an expansive pocket. The imaging shows circumferential flow between the left ventricle and the aneurysm. Additionally, a left-to-right shunt is observed through the interventricular patch (lower arrow). (*B*) The parasternal short-axis view shows the aneurysmal sac with a large neck. (*C*) The apical four-chamber view shows a dehiscent patch (upper arrow). (*D*) The apical four-chamber view exhibits a left-to-right shunt (upper arrow), along with an aneurysmal outpouching in the inferoseptal wall (lower arrow). (*E*) Steady-state free precession short-axis plane revealing an interventricular communication measuring 8 mm. (*F*) Delayed gadolinium enhancement short-axis image illustrating transmural hyperintensity, with no hyperintense signal noted in the pericardium around the left ventricular aneurysm. It also identifies the fibrotic myocardium around the rupture site, which may contribute to patch failure. (*G*) Delayed gadolinium enhancement two-chamber view showing transmural contrast uptake within the left ventricular aneurysm, without evidence of pericardial hyperintensity excluding a pseudo-aneurysm. (*H*) Cine four-chamber view depicting an interventricular communication at the basal inferoseptal wall.

Five years later, he presented with acute congestive heart failure. Echocardiography showed a persistent 8 mm shunt, a stable inferoseptal aneurysm (panel C), dilated right ventricle (44 mm), pulmonary artery pressure of 66 mmHg, and moderate tricuspid regurgitation. Cardiac magnetic resonance imaging confirmed a dehiscent patch with a left-to-right shunt (panel F) and an adjacent aneurysm (31 mm neck, 35 mm width, 40 mm length) without thrombus (*Figure 1*). Left and right end-diastolic volumes were elevated at 192 and 152 mL/m^2^, respectively. Left ventricular ejection fraction was 40%, with impaired right ventricular function. The patient improved with intravenous furosemide. Patch closure combined with aneurysmectomy were proposed but declined. He was discharged on medical therapy including aspirin 75 mg, atorvastatine 10 mg, and furosemide 40 mg once daily along with heart failure quadritherapy. He remained asymptomatic at 1-year follow-up, with stable interventricular shunt and aneurysm.

## Data Availability

All data supporting the findings of this case report are included within the article. Additional details are available from the corresponding author upon reasonable request.

## Notes

**Consent:** Written informed consent was obtained from the patient for publication of this case report and accompanying images. A copy of the written consent is available for review by the Editor-in-Chief of this journal on request.

**Funding:** The authors received no specific funding for this work.

